# Corneal higher order aberrations by Sirius topography and their relation to different refractive errors

**DOI:** 10.1186/s12886-023-02841-4

**Published:** 2023-03-16

**Authors:** Abdelrahman Salman, Obeda Kailani, Marwan Ghabra, Rana Omran, Taym R. Darwish, Rafea Shaaban, Hussein Ibrahim, Hala Alhaji, Hussam Khalil

**Affiliations:** 1 Department of Ophthalmology, Tishreen University, Latakia, Syria; 2 Department of Ophthalmology, King’s College Hospital NHS Foundation Trust, London, UK; 3 Department of Ophthalmology, Whipps Cross University Hospital, London, Leytonstone UK; 4 Department of Ophthalmology, Eye Surgical Hospital, Damascus, Syria; 5 Department of Ophthalmology, Tartous University, Tartous, Syria; 6 Eye Surgical Hospital, Health Ministry, Damascus, Syria

**Keywords:** Zernike coefficients, Corneal Higher order aberrations, Refractive errors, Sirius, Astigmatism

## Abstract

**Purpose:**

To compare the root mean square (RMS) of anterior corneal higher-order aberrations (HOAs) in ametropic and emmetropic eyes.

**Methods:**

This retrospective observational study was conducted at the Department of Ophthalmology, Tishreen University Hospital, Latakia, Syria. Study eyes were divided into four groups based on refractive error: mild-to-moderate myopia, hypermetropia, myopic astigmatism, and emmetropic eyes as controls. The following anterior corneal HOAs were evaluated using the Scheimpflug-Placido Sirius (CSO, Italy) tomographer over 6 mm pupil: Root mean square (RMS) total corneal HOAs, RMS trefoil, RMS coma and RMS spherical aberrations.

**Results:**

RMS values of total HOAs, trefoil and coma showed statistically significant differences in all four groups (*P* < 0.05, all). HOAs were noted to be lowest in the control group (0.18 ± 0.09, 011 ± 0.08 and 0.09 ± 0.08 μm, respectively) and highest in the myopic astigmatism group (0.31 ± 0.16, 0.15 ± 0.12, 0.17 ± 0.14 μm, respectively). RMS spherical aberration was lowest in the astigmatism group (0.00 ± 0.16 μm) with a statistically significant difference from that in the control group (0.05 ± 0.07 μm, *P* = 0.049).

**Conclusion:**

The mean RMS values of total HOAs, trefoil and coma were highest in the astigmatism group and lowest in the control group. However, spherical aberration was minimal in the astigmatism group. A better understanding and targeted treatment of higher-order aberrations in ametropic human eyes, and in particular eyes with astigmatism, may enhance visual quality and performance in the treatment of refractive errors. Recognising atypical HOAs may also assist in the early detection of pathological conditions such as keratoconus.

## Introduction

Higher-order aberrations (HOAs) are small optical irregularities of the eye, which can have an impact on the quality of the retinal image [1]. In contrast to lower order aberrations, these errors cannot be corrected with regular spectacles and most contact lenses. In recent years, advances in ocular diagnostic and treatment modalities have brought corneal higher order aberrations to the forefront of ophthalmologists and optometrists in enhancing visual quality. The ability to measure wavefront aberrations of the eye and incorporation of this data into treatment profiles has led to a significant improvement in the quality of vision following refractive surgery and the ability to correct surgical-induced HOAs in addition to lower order aberrations (sphere and cylinder) [2, 3]. Moreover, the evaluation of HOAs in normal eyes has been demonstrated to be helpful in the early detection of pathological conditions such as keratoconus (KC) [4–6]. More recently, a study by Wallerstein et al. assessed the potential application of corneal HOA excimer ablation map imaging in not only identifying KC, but using this novel diagnostic method in grading KC [7].

Accurate measurements of higher-order aberration are essential to clinical studies evaluating refractive error and image quality [8]. The relationship between HOA and refractive error is of particular interest as it has been suggested that retinal defocus and cues from HOA may affect growth of the eye [9]. Additionally, HOAs are responsible for an array of visual symptoms including dysphotopsia, glare, as well as a reduction in contrast sensitivity following corneal refractive surgery [10]. The cornea accounts for approximately 90% of ocular aberrations [1].

Corneal aberrometry has the ability to evaluate the anterior ocular surface, which is the most effective refractive surface of the eye’s optical system [4]. The Sirius Scheimpflug–Placido Tomographer (Costruzione Strumenti Oftalmici, Florence, Italy) provides anterior and posterior corneal topography, corneal wavefront analysis, as well as corneal pachymetry.

This study aimed to compare anterior corneal HOAs among patients with mild-to-moderate myopia, hypermetropia and myopic astigmatism, highlighting key differences to emmetropic controls, enabling a better understanding of the respective profiles of HOAs.

## Material and methods

This retrospective observational comparative study was conducted at the Department of Ophthalmology, Tishreen University Hospital, Latakia, Syria. Ethical approval for the study was obtained from the University of Tishreen Research Ethics Committee and the research followed the tenets of the Declaration of Helsinki, with written informed consent obtained.

Details of the sampling strategy have been published elsewhere [11]. Normative control data was obtained from recruited volunteers and patients screened prior to refractive surgery. Five-hundred and seventy-three participants were included in the study. A single eye from each participant was included using a simple random sampling. Eyes were divided into four groups according to their refractive errors. Of the total 573 eyes, 104 (18.15%) eyes were emmetropic (controls) and 469 (81.85%) were ametropic. Emmetropic eyes consisted of those with manifest refraction spherical equivalent (MRSE) within ± 0.5 D. All emmetropic subjects had normal corneal topography in both eyes with uncorrected distance visual acuity (UDVA) of 20/20 or better. Ametropic eyes were divided into three groups according to their refractive errors: Mild to moderate myopia (-1.0 ≤ RE ≤ -6.0) (211 eyes), simple myopic astigmatism (cylinder ≤ -1D) (175 eyes), and hypermetropes (+ 1.00 ≤ RE ≤  + 4.00D) (83 eyes). Only ametropic eyes with corrected distance visual acuity (CDVA) of 20/20 or better were included.

### Exclusion criteria

Patients were excluded if they had previous ocular surgery (cataract, glaucoma, corneal cross-linking, excimer laser surgery, intrastromal corneal rings, phakic intraocular lens). Ocular conditions which could also confound corneal HOAs were also excluded (amblyopia, ocular surface inflammation, dry eye disease, KC, KC-suspect, corneal dystrophies, or intraocular inflammation). Systemic autoimmune diseases, current or recent pregnancy (within 1 year) and active lactation were also excluded. Prior to evaluation, contact lens-wearing patients were asked to discontinue wearing their lenses for 3 weeks and 1 week for rigid and soft contact lenses, respectively.

### Clinical assessment and evaluation

Evaluation included the measurement of UDVA, CDVA, MRSE, slit-lamp biomicroscopy, retinoscopy and fundoscopy. Cycloplegic refraction was induced with three drops of 1% cyclopentolate hydrochloride (Cyclomed 1% ophthalmic solution; Medico Pharmaceutical Co., Ltd., Syria) at 5-min intervals, and auto-refractometry was done 60 min after the first instillation using an auto ref-keratometer (SEIKO CO, GR-3500KA, Japan). Adequate pupil dilation and unresponsiveness to photopic stimuli were confirmed prior to conducting measurement. Three measurements were recorded for each eye and averaged for the analyses.

**Fig. 1:**
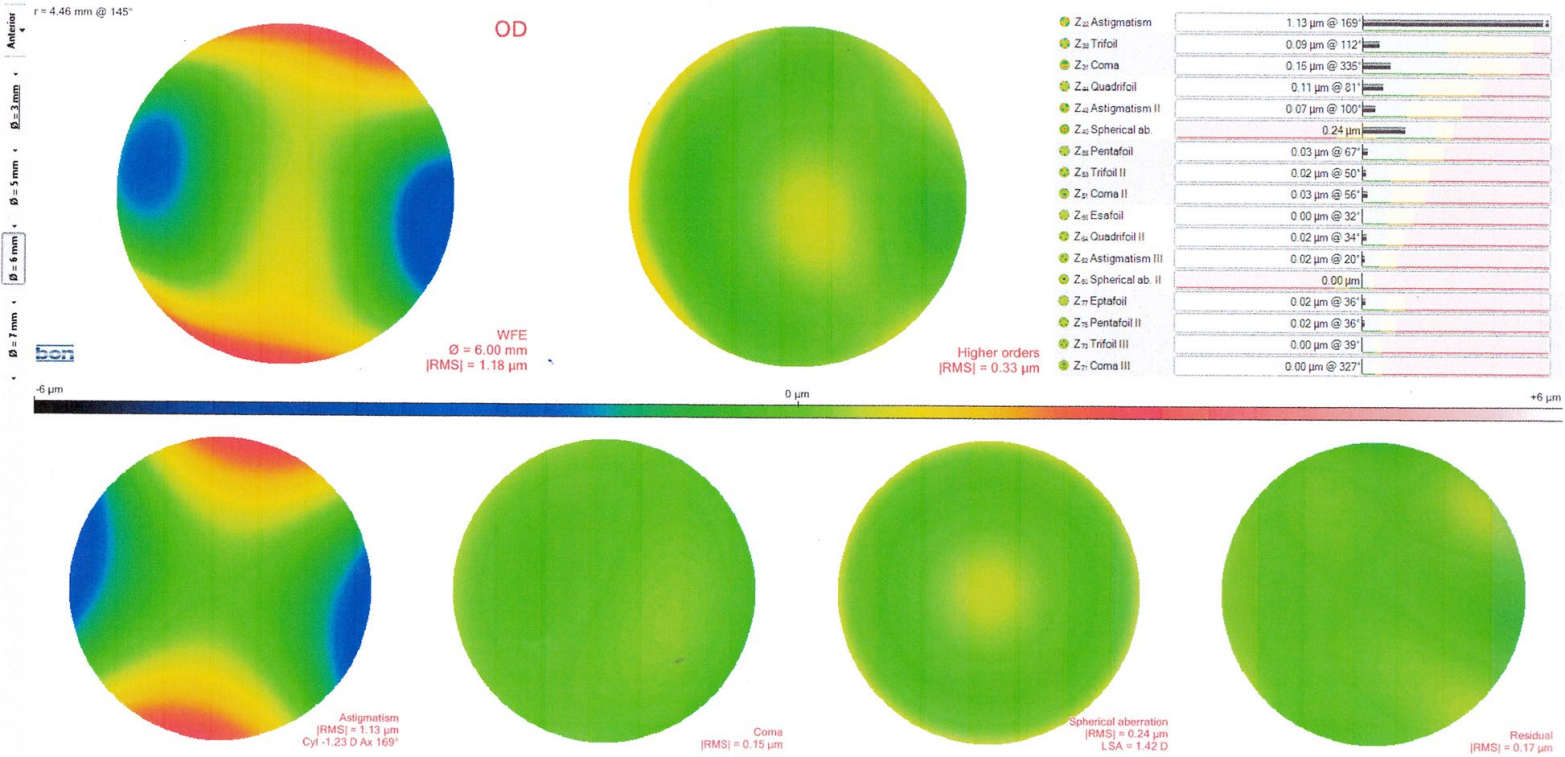
Sirius aberrometer output display for anterior corneal-higher order aberrations

Corneal topography and corneal aberrometry were obtained using the Scheimpflug-Placido topographer (Sirius, Phoenix software v.2.6.4.44 Costruzione Strumenti Oftalmici, Florence, Italy). The accuracy of the Sirius corneal topographer has been previously reported [12]. Anterior corneal higher order aberrations were measured at the central 6.0 mm zone. Three well-focused, aligned and centred images were obtained for each eye. To optimise image capture, patients were asked to blink before each image capture to eliminate the effect of corneal surface dryness. Placido disc mires up to the 17^th^ ring had to be continuous to consider videokeratography of adequate quality and satisfactory for calculation of the Zernike coefficients (Zm,n) for a 6.0 mm simulated pupil [13]. Software acquisition was uniform for all data points for consistency. Figure 1 illustrates a sample of Sirius HOAs values.

The collected HOAs data included: Root mean square (RMS) total HOAs up to the 7th Zernike order, RMS of third-order trefoil Z (3, ± 3) (square root of the sum of squared coefficients of vertical trefoil Z3,-3 and horizontal trefoil Z3,3), RMS of third-order coma Z (3, ± 1) (square root of the sum of squared coefficients of vertical coma Z3,-1 and horizontal coma Z3,1), and RMS of fourth order spherical aberration Z (4, 0) (square root of the sum of squared coefficients of Z4, 0).

### Statistical analysis

Quantitative data were represented as mean and standard deviation. The Shapiro–Wilk test was used to assess the conformity of the data to normal distribution. When the data were normally distributed, the one-way analysis of variance (ANOVA) test and the Student's t-test were used to compare variables of four and two groups, respectively. In cases of non-normal distribution, the Kruskal–Wallis test and Mann–Whitney test were used to compare four and two groups, respectively. The Spearman correlation coefficients were used to further elucidate the relationship between refractive errors and corneal aberrations. A *P* value of < 0.05 was considered significant.

## Results

Demographic and refractive data for each group are presented in Table 1. A total of 573 eyes of 573 subjects were included in this study. Three hundred and forty-nine (60.91%) subjects were female and 224 (39.09%) subjects were males.

**Table 1:** Demographic and refractive data of all groups

	**Study groups**			
	Control	Myopia	Astigmatism	Hypermetropia
**N**	104	211	175	83
**Sex (M/F)**	44/60	79/132	77/98	24/59
**Refractive errors (Mean ± SD)**				
** Sphere**	-0.04 ± 0.25	-1.72 ± 1.38	-1.10 ± 2.87	1.52 ± 1.37
** Cylinder**	-0.49 ± 0.20	-0.50 ± 0.18	-1.56 ± 1.42	-0.40 ± 0.39
** Spherical equivalent**	-0.25 ± 0.22	-1.91 ± 1.40	-1.78 ± 2.98	1.38 ± 1.46

*N* number, *F* female, *M* male, *SD* standard deviations

The subjects’ ages ranged from 18 to 40 years with a mean age of 23.0 ± 4.90 years. The mean age of controls was 21.49 ± 2.33 years, while the other three ametropic cohorts were 22.74 ± 3.70, 23.11 ± 5.46 and 25.27 ± 7.27 years for myopia, myopic astigmatism and hypermetropia, respectively. The mean age revealed statistically significant differences between the control and astigmatism groups, between the control and hypermetropia groups, between the myopia and hypermetropia groups, and between the astigmatism and hypermetropia groups (*P* < 0.05, for all). The ANOVA test revealed a statistically significant difference between the four groups (*P* = 0.0001). Age differences between the study groups are presented in Table 2.

**Table 2:** Age distribution of patients in study groups

Study group	Age (mean ± SD)	Study group comparison	*P* value
Control	21.49 ± 2.33	Control / Myopia	0.188
Myopia	22.74 ± 3.70	Control / Astigmatism	0.041^*^
Astigmatism	23.11 ± 5.46	Control / Hypermetropia	0.0001^*^
Hypermetropia	25.27 ± 7.27	Myopia / Astigmatism	0.37
		Myopia / Hypermetropia	0.0001^*^
		Astigmatism / Hypermetropia	0.005^*^
		ANOVA	0.0001^*^

*SD* standard deviations. ANOVA test was used to compare differences of means among the four groups. Student's t-test was used to compare the difference between means of each two samples

^*^Reached statistical significance (*P* < 0.05)

The mean and standard deviation of HOAs of anterior corneal aberrations for all groups are shown in Table 3. Statistical analyses of anterior corneal HOAs in the different groups of refractive errors revealed that the mean RMS values of total HOAs, trefoil and coma were at their lowest levels in the control group (0.18 ± 0.09, 0.11 ± 0.08 and 0.09 ± 0.08 μm, respectively). In contrast, the mean RMS spherical aberrations value was highest in the control group (0.05 ± 0.07 μm). Anterior corneal aberrations were compared using the Kruskal–Wallis test. RMS total HOAs, RMS trefoil and RMS spherical aberrations were significantly different between groups (*P* < 0.05, for all).

**Table 3:** RMS ± standard deviations of anterior corneal higher-order aberrations of all groups

	The study groups				
	Control	Myopia	Astigmatism	Hypermetropia	*P*-value
	Mean ± SD	Mean ± SD	Mean ± SD	Mean ± SD	
RMS total HOAs	0.18 ± 0.09	0.24 ± 0.14	0.31 ± 0.16	0.26 ± 0.17	0.0001^*^
RMS trefoil	0.11 ± 0.08	0.11 ± 0.07	0.15 ± 0.12	0.12 ± 0.08	0.0001^*^
RMS coma	0.09 ± 0.08	0.12 ± 0.11	0.17 ± 0.14	0.15 ± 0.15	0.0501
RMS Spherical Ab	0.05 ± 0.07	0.03 ± 0.14	0.00 ± 0.16	0.03 ± 0.14	0.0136^*^

The *p*-value tests any significant difference of HOAs variables between the four groups using the Kruskal–Wallis test

*RMS* Root Mean Square, *N* number, *SD* standard deviation, *Ab* Aberrations

^*^Reached statistical significance (*P* < 0.05)

**Fig. 2:**
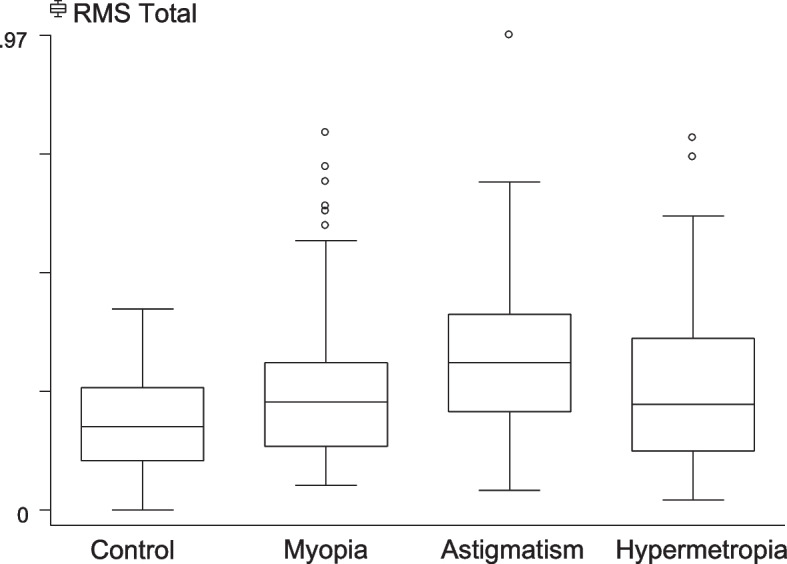
RMS Total HOAs distribution among different groups

The Mann–Whitney test was used to compare the difference between the means of each two samples (Table 4). The highest level of RMS total HOAs value (0.31 ± 0.16) was seen in the astigmatism group (Fig. 2), with statistically significant differences between the control and myopic eyes, between the control and astigmatic eyes, between the myopic and astigmatic eyes, and between the astigmatic and hypermetropic eyes (*P* < 0.05, for all).

**Table 4:** Difference of anterior corneal HOAs among each of the study group pairs

HOAs by refractive errors	***P*-value**					
	**Control/ Myopia**	**Control/ Astigmatism**	**Control/ Hypermetropia**	**Myopia/ Astigmatism**	**Myopia/ Hypermetropia**	**Astigmatism/ Hypermetropia**
Total HOAs	**0.0100**	**0.0001**	**0.0050**	**0.0001**	1.00	**0.0340**
Trefoil	1.0000	**0.0020**	1.0000	**0.0000**	1.0000	**0.0240**
Coma	0.2070	**0.0000**	**0.0210**	**0.0020**	0.9490	0.9630
Spherical aberrations	1.0000	**0.0490**	1.0000	0.5810	1.0000	0.6850

*HOAs *higher-order aberrations

Mann–Whitney test was used to compare the difference between means of each two samples. Values in bold reached statistical significance (*P* < 0.05)

**Fig. 3:**
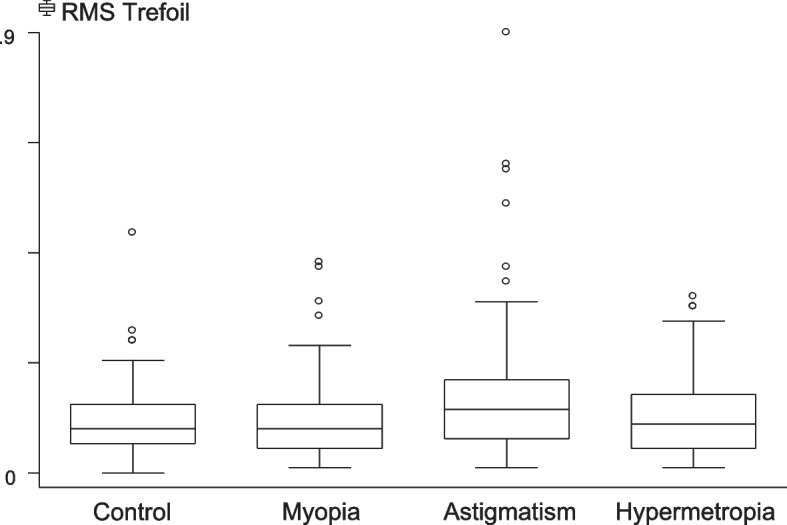
Trefoil distribution among different groups

RMS trefoil was highest (0.15 ± 0.12) in the astigmatism group (Fig. 3), with statistically significant differences when compared between the control and astigmatic eyes, between the myopic and astigmatic eyes and between the astigmatic and hypermetropic eyes (*P* < 0.05, for all).

**Fig. 4:**
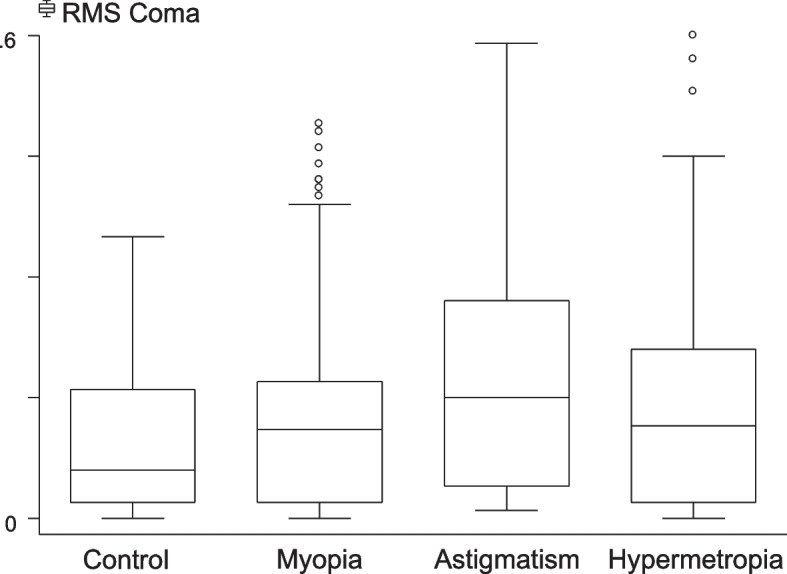
Coma distribution among different groups

RMS coma was also highest (0.17 ± 0.14) in the astigmatism group (Fig. 4), with statistically significant differences between the control and astigmatism groups, between the control and hypermetropia groups, and between the myopia and astigmatism groups (*P* < 0.05, for all).

**Fig. 5:**
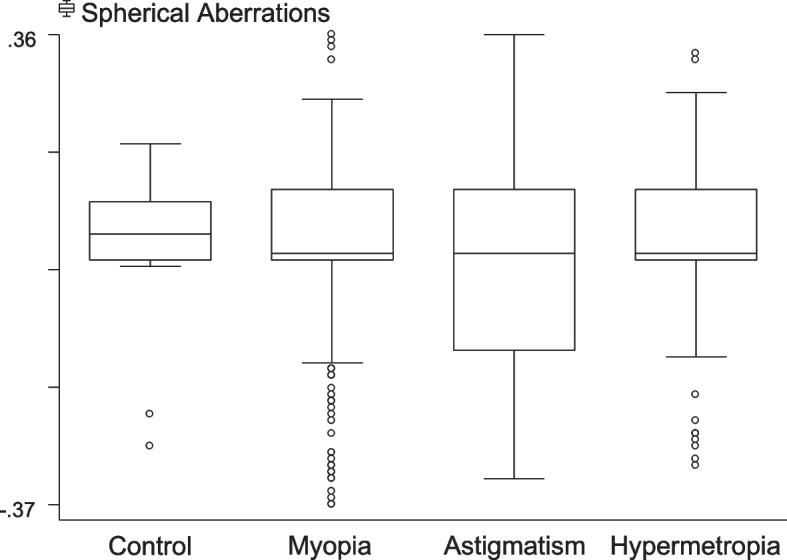
Spherical aberrations distribution among different groups

Figure 5 demonstrates the spherical aberration findings, showing the lowest levels (0.00 ± 0.16) in the astigmatism group and the highest in the control group. However, spherical aberration was significantly different only between the control and astigmatism groups (*P* = 0.049).

Table 5 shows Spearman correlation between each type of refractive error and the amount of HOAs. Myopia showed a statistically significant negative correlation with total HOAs (*r* = -0.1381, *P* = 0.0484), whereas hypermetropia showed a positive correlation with RMS total HOAs (*r* = 0.4864, *P* < 0.0001) and coma (*r* = 0.5106, *P* < 0.0001), and a statistically significant negative correlation with RMS spherical aberrations (*r* = -0.2721, *P* = 0.0174).

**Table 5:** Spearman correlation between type of refractive errors and HOAs

Refractive error		Total HOAs	Coma	Trefoil	Spherical aberrations
**Myopia**	*r*	-0.1381	-0.0785	-0.0489	0.0917
	*p* value	0.0484^*^	0.2632	0.4865	0.1908
**Astigmatism**	*r*	-0.1453	-0.0887	-0.1384	0.0461
	*p* value	0.0609	0.2560	0.0753	0.5539
**Hypermetropia**	*r*	0.4864	0.5106	0.1169	-0.2721
	*p* value	< 0.0001^*^	< 0.0001^*^	0.3114	0.0174^*^

*HOAs* higher-order aberrations, *r* correlation coefficient

^*^Reached statistical significance (*P* < 0.05

The Spearman correlation between HOAs and age, and between HOAs and refractive errors (sphere, cylinder, spherical equivalent) are shown in Table 6. Cylinder revealed a statistically significant negative correlation with RMS total HOAs (*r* = -0.2173, *P* < 0.0001), trefoil (*r* = -0.2034, *P* < 0.0001), and coma (*r* = -0.1221, *P* = 0.0093). Spherical equivalent revealed a statistically significant negative correlation with total HOAs (*r* = , *P* < 0.0001). While Both RMS total HOAs (*r* = 0.1649, *P* = 0.0001) and RMS coma (*r* = 0.142, *P* = 0.001) showed statistically significant positive correlations with age, spherical aberrations (*r* = -0.0848, *P* < 0.0001) had a negative correlation with age.

**Table 6:** Spearman correlation between HOAs and age and HOAs and amounts of refractive errors (sphere, cylinder, and spherical equivalent)

		**Age**	**Sphere**	**Cylinder**	**Spherical equivalent**
**Total HOAs**	*R*	0.1649	-0.0714	-0.2173	-0.1237
	*p* value	0.0001^*^	0.1154	< 0.0001^*^	0.0041^*^
**Trefoil**	*R*	0.0591	-0.0517	-0.2034	-0.0735
	*p* value	0.1730	0.2542	< 0.0001^*^	0.0889
**Coma**	*R*	0.1420	-0.0335	-0.1221	-0.0622
	*p* value	0.0010^*^	0.4614	0.0093^*^	0.150
**Spherical Ab**	*R*	-0.0848	0.0267	-0.0131	0.0403
	*p* value	< 0.0001^*^	0.5556	0.7806	0.3514

*HOAs* higher-order aberrations, *Ab* aberration, r: correlation coefficient

^*^Reached statistical significance (*P* < 0.05

## Discussion

In our study, we investigated corneal HOAs in emmetropic, myopic, hypermetropic and astigmatic eyes. We had a particular focus on corneal higher order aberrations that are most relevant to clinical practice, namely coma, trefoil and spherical aberrations [13]. While several studies evaluated total ocular HOAs in patients with refractive errors, there are few similar studies that evaluate corneal HOAs [11, 14, 15]). To the best of our knowledge, no study has yet reported the distribution of HOAs in the Syrian population.

Some studies found no significant differences among different refractive groups in terms of aberrations [16–18], while other studies showed that myopic patients have more aberrations than astigmatic and hypermetropic patients [19, 20]). Kasahara et al. evaluated HOAs in patients with myopia and found that highly myopic eyes had more HOAs than emmetropic eyes due to the increased internal aberrations [21]. Our results showed no statistically significant differences in coma, trefoil and spherical aberrations between myopic and emmetropic eyes. However, the discrepancy in findings could be attributed to the fact that our study evaluated corneal HOAs in low to moderate myopia while Kasahara et al. evaluated ocular (internal and corneal) HOAs in highly myopic eyes. Mohammadpour et al. reported an increase in HOAs, correlating with an increase in ocular astigmatism [22]. Similarly, Zhang et al. found that an increase in astigmatism was associated with an increase in coma, trefoil and total HOAs [23]. These findings were similar to our results, where we also found that total HOAs, coma and trefoil were at their highest levels in the astigmatism group.

Total HOAs in our study was comparable to an Egyptian study by Anbar et al. where they used the Scheimpflug–Placido topography (Sirius, CSO, Italy) to obtain corneal HOAs in 750 patients with mild-to-moderate myopia, high myopia, hypermetropia, simple myopic astigmatism, and simple hypermetropic astigmatism [12]. They found that total HOAs were highest (0.99 ± 0.70 μm) in hypermetropes. In their prospective study, Khan et al. used a Wavelight Allergo Analyzer to evaluate ocular HOAs in 200 eyes of 121 patients with different refractive errors (myopia, astigmatism and hypermetropia) [24]. Their results showed that total HOAs were highest ( 0.96 ± 0.96) in hypermetropic patients. In contrast, our results showed that total HOAs were at their highest level (0.31 ± 0.16 μm) in the myopic astigmatism group. A possible explanation for the contradiction may be due to the variety of sample size utilizations and research methodologies.

In concordance with the previous report [25]), we observed that total HOAs (0.26 ± 0.17 μm) in hypermetropic eyes were mainly due to coma aberrations (0.15 ± 0.15 μm). The angle kappa is known to be larger in hypermetropes compared to myopes and emmetropes, and a larger displacement of the pupillary axis from the visual axis is responsible for higher levels of coma in hypermetropic eyes [26]. Our findings were consistent with this, as we found that coma aberration was significantly higher in hypermetropic eyes when compared to emmetropic and myopic eyes. Furthermore, our results showed that the group with the highest cylinder is the group with the highest RMS coma. The corneal/refractive cylinder magnitude correlates to higher-order aberrations (e.g. coma). This agrees with a previous study where the authors also found that eyes with high astigmatism tend to have higher higher-order aberrations, such as coma [27].

Little et al. demonstrated that spherical aberration was significantly related to axial length (but not refractive error), with longer eyes having less positive values of fourth order and RMS spherical aberration [10]. Zhao et al. found that spherical aberrations of astigmatic corneas were similar to those of non-astigmatic corneas [28]. Interestingly, our results showed that spherical aberrations were at their lowest level (0.0 ± 0.16 μm) in astigmatic eyes. He et al. used a ray-tracing technique to investigate HOAs aberrations in adult patients with refractive errors and found higher spherical aberrations in myopic eyes as compared to emmetropic eyes [19]. Marcos observed that intraocular spherical aberrations became more negative in high myopia due to the crystalline lens [29]. Our results revealed positive spherical aberrations values in all groups. Studying HOAs in 675 adolescents, Philip et al. found no significant difference in corneal spherical aberrations among the myopic, hypermetropic and emmetropic groups [30]. These findings are consistent with ours, as spherical aberrations were not significantly different between emmetropic, myopic and hypermetropic groups.

Contrary to He et al. who reported no significant relation of myopia with HOAs [20], Khan et al. reported a statistically significant negative correlation of hypermetropia with RMS of total HOAs and spherical aberration [24]. In contrast, our results showed a statistically significant positive correlation `r` of hypermetropia with RMS of total HOAs and coma, and a statistically significant negative correlation of hypermetropia with RMS spherical aberrations. Amano et al. investigated age-related changes in ocular and corneal higher-order wavefront aberrations where they found that both corneal and ocular coma RMS were positively correlated with age [31]. These findings are consistent with ours, confirming that corneal coma increases with age.

The lack of consensus in the literature comparing HOAs and refractive error may exist due to a variety of reasons. Extensive variation in sample sizes, differing classifications of refractive error, differences in subject age, ethnicity, and data acquisition methodology, or a combination of these factors may account for the disparity in findings. Unfortunately, most of the studies reported in the literature are retrospective, so prospective multicentre studies with a larger sample size are needed to confirm these findings.

One key limitation of this study was that the distribution of refractive error groups was not homogeneous. Since our study included population-based randomized subjects, differences between the sample numbers across different refractive error groups could not be avoided. The absence of highly myopic subjects and hyperopic astigmatism groups was also considered as another limitation of our study. Moreover, the retrospective nature of the study had implications, prompting the need for further prospective multi-centre evidence to substantiate the evidence-base. To the best of our knowledge, this study was the first to investigate the distribution of HOAs in patients with refractive error in a Syrian population.

## Conclusion

Our findings confirmed that there was a significant difference in corneal higher-order aberrations between different refractive groups. Notably, the mean RMS values of total HOAs, trefoil and coma were highest in astigmatic eyes and lowest in emmetropic eyes. Spherical aberration was minimal in the astigmatism group. Results of this study must be considered in diagnostic and therapeutic refractive procedures. Knowledge of the distribution of higher-order aberrations in the normal population can be helpful to achieve a better quality of vision and prevent significant HOA increase following refractive surgery. We recommend that HOAs should be evaluated in refractive surgery candidates and treatment considerations should be taken by factoring these in. In addition, knowledge of the normal values of HOAs in the normal population is useful and may contribute to the arsenal of diagnostic assessment, particularly in the early diagnosis of pathological conditions such as keratoconus.

## Data Availability

The datasets generated and analysed during the current study are not publicly available due to containing information that could compromise the privacy of research participants but are available from the corresponding author (AS) on reasonable request.
